# Downregulation of Aquaporin 3 Mediated the Laxative Effect in the Rat Colon by a Purified Resin Glycoside Fraction from Pharbitis Semen

**DOI:** 10.1155/2019/9406342

**Published:** 2019-01-13

**Authors:** Dongrong Zhu, Chen Chen, Lijuan Bai, Lingyi Kong, Jianguang Luo

**Affiliations:** Jiangsu Key Laboratory of Bioactive Natural Product Research and State Key Laboratory of Natural Medicines, School of Traditional Chinese Pharmacy, China Pharmaceutical University, Nanjing 210009, China

## Abstract

**Background:**

Pharbitis Semen, the seeds of* Pharbitis nil*, is widely used as a traditional purgative medicine in China, Korea, and Japan. This study investigated the laxative effects of a purified resin glycoside fraction obtained in our previous study from Pharbitis Semen in vivo and in vitro.

**Materials and Methods:**

After orally administering a purified resin glycoside fraction from Pharbitis Semen (RFP) to rats, the content of fecal water, AQP3, NF-*κ*B, COX-2 expression, and the prostaglandin E_2_ (PGE_2_) concentrations in the colon were examined. Moreover, human intestinal epithelial cells (HT-29) were used to investigate the mechanism of RFP decreasing the AQP3 expression.

**Results:**

Results obtained showed that treatment with RFP increased the feces excretion and fecal water content of rats in a dose-dependent manner. More interestingly, AQP3 expression was suppressed by RFP treatment both in the rat colons and in HT-29 cells, while the NF-*κ*B pathway-mediated PGE_2_ production was activated. Interestingly, pretreating rats with BAY-11-7082 (NF-*κ*B inhibitor) or indomethacin (COX-2 inhibitor) and RFP neither induced diarrhea nor decreased the AQP3 expression in the colon.

**Conclusions:**

The purgative property of the purified resin glycoside fraction was attributed to NF-*κ*B activation in the colon, which increased the COX-2-mediated secretion of PGE_2_. PGE_2_ decreased AQP3 expression which inhibits water absorbed from the intestine to the blood vessel side, resulting in the laxative effect of RFP.

## 1. Introduction

Aquaporins (AQPs) are membrane proteins that function as water/glycerol channels and play a vital role in water transport across cell membranes. To date, 13 types of AQPs channels have been identified [1]. There are 9 types of AQP in the intestine, such as AQPs: 1-4 and AQPs: 7-11 [2, 3]. Among them, AQP3 is the most important aquaporin of the colon. It is located in the colonic villus epithelial cells and contributed to the transport of water [4]. It has been reported previously that many factors could lead to laxative effect through altering AQP3 expression to prevent the water transport in colon, such as gut hormone, 5-hydroxytryptamine, bacterial pathogens, and laxative MgSO_4_ [5–7].

Resin glycosides, as the characteristic constituents of Convolvulaceae, are reported to be responsible for the drastic purgative behavior of all the important morning glory family species used in traditional medicines. Pharbitis Semen, the seeds of* Pharbitis nil *(Convolvulaceae), are widely used to treat constipation [8]. Previous studies only proposed that Pharbitis Semen triggered diarrhea through accelerating peristalsis in the colon resulting in water elimination [9]. However, the material basis and molecular mechanism involved in the laxative effect of Pharbitis Semen have not yet been elucidated. Many studies have demonstrated that aquaporins (AQPs) mediated the cathartic effect of laxatives [5, 10, 11]. The nuclear factor-kappa B (NF-*κ*B) is important in regulating cellular responses. There are many known NF-*κ*B pathway activators such as stress, activation of oncogenes and kinases, cytokines, and dysregulation of cell receptors [12]. It was reported that the expression of AQP3 in the rat colon was downregulated via transcription factors NF-*κ*B activation [13]. Another research reported that PGE_2_, a synthetic product of COX-2, is of vital importance for AQP3 regulation to generate the laxative effect in rhubarb extract [14]. We hypothesized that the laxative effect of Pharbitis Semen may be related to AQP3 regulation in the colon mediated by NF-*κ*B activation and possibly involvement of PGE_2_ production.

In this study, we investigated the material basis of Pharbitis Semen induction of diarrhea and explored the mechanism of a purified resin glycoside fraction from Pharbitis Semen (RFP) on AQP3 protein expression in vitro and in vivo.

## 2. Materials and Methods

### 2.1. Extraction and Isolation of the Purified Resin Glycoside Fraction (RFP)

The purified resin glycoside fraction from Pharbitis Semen (RFP) was obtained from our previous work [15]. The MeOH-insoluble fraction (Fr.C') was derivatized by NH_2_ silica gel on-column catalyzation to obtain individual constituents. Eleven acylated resin glycosidic acid methyl esters were obtained in this fraction [15]. Therefore, Fr.C was characterized as a resin glycoside fraction from Pharbitis Semen (RFP).

### 2.2. Materials

RFP was dissolved in carmellose sodium (CMC-Na) or dimethyl sulfoxide (DMSO) before the administration of rats or cells, respectively. Indomethacin and MgSO_4_ were obtained from Aladdin (Shanghai, China). BAY 11-7082 (BAY) was purchased from MCE (HY-13453, NJ, USA). Prostaglandin E_2_ (PGE_2_) was purchased from Santa Cruz Biotechnology (sc-201225A, TX, USA). PGE_2_ ELISA kit was purchased from R&D Systems, Inc. (St. Louis, MO, USA). The following primary antibodies were used: AQP3 (ab125219, Abcam, Cambridge, UK), COX-2 and p-p65 (Cell Signaling Technology, Boston, USA), Glyceraldehyde Phosphate Dehydrogenase (GAPDH) and Proliferating Cell Nuclear Antigen (PCNA) (Beyotime, Nanjing, China), and mouse IgG HRP and Alexa Fluor® 647 Goat Anti-Rabbit IgG (Fcmrcs, Nanjing, China).

### 2.3. Animals

Nine-week-old male specific pathogen-free SD rats (220-250 g) were obtained from the Experimental Animal Center of Yangzhou University (Yangzhou, China). Keep these animals in climate-controlled facilities with automatic light and dark cycles and allow free access to water and standard food. It is kept and treated in strict accordance with the obligations of the Animal Ethics Committee of China Pharmaceutical University and the guidelines for the care and use of laboratory animals of the National Institutes of Health.

### 2.4. Animals Treatment

The rats were divided separately into two groups randomly. The first group (N1 = 30) was divided separately into five small groups with the equal number: the normal control group, the RFP (31.25, 62.5, and 125 mg/kg, respectively)-treated groups, and MgSO_4_ (2 g/kg)-treated group. RFP or MgSO_4_ was orally administered to the five group rats (water was provided ad libitum) after fasting for 18 h. The second group (N2=24) was divided separately into four small groups (vehicle, RFP-treated vehicle, BAY, or indomethacin combined with RFP administration group). BAY (10 mg/kg) or indomethacin (20 mg/kg) was intraperitoneally administered to rats 1 h before RFP treatment. After different treatment for 6 h, all these rats were sacrificed and their colons were removed. The colon was washed with PBS and then rapidly frozen with liquid nitrogen and stored at -80°C. Fecal samples were disposed as described formerly [14]. The water content of feces was calculated based on the difference between wet and dry fecal weights.

### 2.5. Immunohistochemistry and Histological Examination of Rat Intestine

Rats were sacrificed at 6 h after RFP administration. The colons were removed and fixed immediately for 3 h in 4% paraformaldehyde after washing with PBS. Three tissue samples of every group dissected from colon were used. Procedures of immunohistochemistry and hematoxylin and eosin (H&E) staining were carried out as formerly described [11, 16]. Tissue slices were visualized using NanoZoomer 2.0 RS (Beijing, China).

### 2.6. Cell Culture

HT-29 cells were purchased from the Cell Bank of Shanghai Institute of Biochemistry and Cell Biology, Chinese Academy of Sciences (Shanghai, China). This cell line was cultured in PRMI-1640 with 10% fetal bovine serum (FBS, GIBCO, USA), 100U/ml penicillin, and 100mg/ml streptomycin at 37°C with 5% CO2.

### 2.7. Cell Viability Assay

Cell viability was measured by MTT assay which has been described previously [11]. Cells were administrated with a series of concentrations of RFP for 6 h. Cell viability was calculated using the following formula: %Cell viability=A1/A0 x100. A1 and A0 represent the absorbance values of the RFP treatment and control, respectively.

### 2.8. Immunofluorescence Assay

Immunofluorescence assay was done according to the procedures used before [11]. After staining, the cells images were analyzed by ImageXpress® Micro Confocal (Molecular Devices, USA).

### 2.9. Prostaglandin E_*2*_ Measurement

Prostaglandin E_2_ (PGE_2_) in the tissue of rats colon or cell supernatant was measured using the PGE_2_ ELISA kit. The PGE_2_ extraction in the colon or cell supernatant was performed according to the manufacturer's protocol included in the PGE_2_ ELISA kit. The content of PGE_2_ in the samples was estimated from the standard curve generated using known concentrations of PGE_2_.

### 2.10. Preparation of Tissue and Cell Protein for Western Blot Analysis

The removed colons from rats at 6 h after the administration of RFP or MgSO_4_ were used. Since AQP3 is mainly expressed in the plasma membrane and the surface of cells, the crude fraction mainly containing the AQP3 is prepared as formerly reported [11].

The protein of AQP3 in HT-29 cells was also extracted. Briefly, RFP-treated HT-29 cells were collected and suspended in RAPI dissecting buffer. The fraction containing AQP3 was obtained as procedures used before [17].

The phosphorylation of p65 is primarily translocated into the nucleus. Therefore, the nuclear fraction was prepared using a nuclear and cytoplasmic protein extraction kit (Beyotime, Nanjing, China) according to the manufacturer's protocol. Cells and tissues were treated with cytoplasmic extraction buffer and placed on ice for 5 minutes. It was then homogenized and centrifuged (4000 x g, 4°C, 10 minutes). After discarding the supernatant, a nuclear extraction buffer was added to the particles. The suspension was centrifuged (16,000 × g, 4°C for 30 minutes) to obtain a supernatant as a core portion. Other proteins were extracted by lysis in RIPA buffer to obtain total protein. The sample was then kept on ice for 30 minutes and then centrifuged at 15,000 g for 10 minutes at 4°C.

All these protein concentrations extracted from tissue or cell were determined using a BCA protein assay kit. Western blot analyses were conducted according to the instruction manual included in the primary antibodies kit. The protein bands were detected using the ChemiDOC™ system (Bio-Rad, Hercules, CA).

### 2.11. Statistical Analysis

The numerical data are expressed as the means ± standard deviation and performed in triplicate. Data from multiple groups were analyzed by one-way ANOVA, followed by Tukey's Multiple Comparison Test. For all the tests, the level of significance was *∗* P < 0.05, *∗∗*P < 0.01, and *∗∗∗* P < 0.001 and “ns” represented that there is no significance.

## 3. Results

### 3.1. RFP Induced Diarrhea in Rats

**Figure 1:**
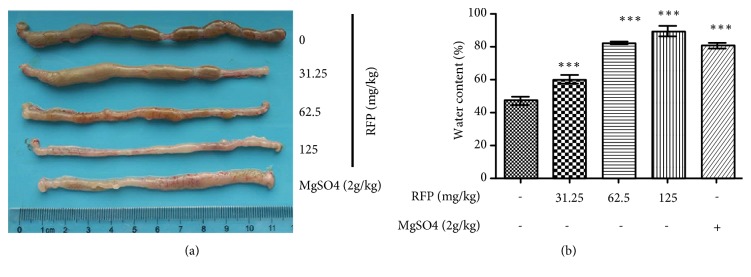
RFP induced diarrhea in rats. (a, b) Oral administration of various doses of RFP (31.25, 62.5 and 125 mg/kg) to rats or MgSO_4_ (2g/kg) as positive control. (a) After 6 h administration, rats were autopsied after sacrifice, macroscopic image of the colon showed that watery stool was obvious. (b) Rat stool samples were collected 6 hours after RFP administration and fecal water content was measured. *∗∗∗* P < 0.001 vs control group.

As shown in Figure 1(a), macroscopic image of colon was observed. The stool in RFP-treated rats (31.25-125 mg/kg) was markedly less than the normal control rats, suggesting severe diarrhea happened after RFP treatment. In addition, the fecal water content after RFP administration increased in a dose-dependent manner (Figure 1(b)). The laxative effect of positive comparison group (MgSO_4_/2 g/kg) is equal to the group of middle dose of RFP (62.5 mg/kg), suggesting that RFP had effective laxative activity.

### 3.2. RFP Decreased the Expression of AQP3 in the Colon of Rats

**Figure 2:**
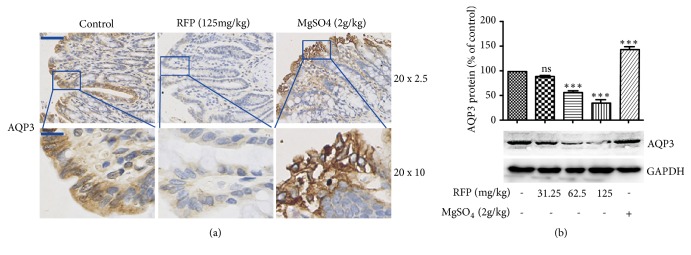
RFP decreased the expression of AQP3 in the colon of rats. (a, b) The colon was removed from the rats 6 hours after RFP or MgSO4 administration. (a) The expression of AQP3 in the RFP-treated rat colons was detected by immunohistochemistry. The positive expression was shown as brown. (20 x 2.5) represents the lower magnification image, bars: 100 *μ*m and (20 x 10) represents amplifying image, bars: 25 *μ*m. (b) Western blotting was done to analyze the protein expression levels of AQP3. *∗∗∗* P < 0.001, ns vs control group.

Changes in protein expression of AQP3 were observed in immunohistochemistry, which exists mainly in mucosal epithelial cells of the colon. An obvious decrease in the expression of AQP3 after RFP administration was observed (Figure 2(a)). Western blotting further confirmed this result (Figure 2(b)). Altogether, these results revealed that RFP decreased AQP3 expression in the mucosal epithelial cells of rat colon.

### 3.3. RFP Activated NF-*κ*B Pathway and Their Downstream Proteins

**Figure 3:**
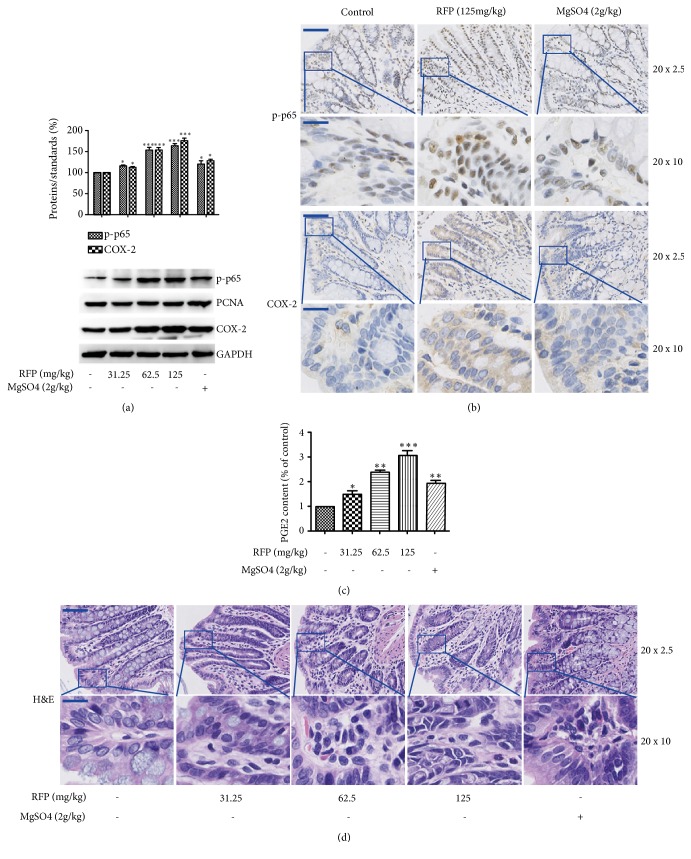
RFP activated NF-*κ*B pathway and their downstream proteins. (a) The expressions of phosphorylated NF-*κ*B (p-p65) in nuclear fractions and cytosolic COX-2 were subjected to western blot analysis. PCNA and GAPDH were used as loading controls of nuclear fraction and cytosolic fraction respectively. *∗*P < 0.05, *∗∗∗*P < 0.001 vs control group. (b) Immunohistochemical technique was used to analyze the expression of p-p65 and COX-2 in the colon. The p-p65 and COX-2 positive result presents brown, low resolution (20 x 2.5, bars: 100 *μ*m) and amplifying image (20 x 10, bars: 25 *μ*m). (c) The PGE_2_ content was detected using the ELISA method. The control group was indicated as 100%. The data represented the means ± SDs for six rats. *∗*P < 0.05, *∗∗*P < 0.01, *∗∗∗*P < 0.001 vs control group. (d) The rats colons after RFP treatment were stained with H&E, low resolution (20 x 2.5, bars: 100 *μ*m) and amplifying image (20 x 10, bars: 25 *μ*m).

Since NF-*κ*B is involved in various kinds of diarrhea, NF-*κ*B activation has been reported to be important to the downregulation of the AQP3 channel [16, 18]. The translocation of NF-*κ*B into the nucleus and downstream protein COX-2 were analyzed. As shown in Figure 3(a), RFP induced NF-*κ*B phosphorylation significantly, indicating the activation of NF-*κ*B signal in rat colon. Similarly, RFP increased the expression of COX-2, which plays an important role in the regulation of intestinal ion secretion and barrier integrity of the colon through the action of its product prostaglandin E_2_ (PGE_2_). In addition, immunohistochemistry also showed the similar results. RFP treatment increased NF-*κ*B nuclear translocation and the COX-2 activation (Figure 3(b)). To further confirm this result, the production of PGE_2_ was detected. RFP administration actually increased the level of PGE_2_ in the colon (Figure 3(c)). These results showed that RFP could activate NF-*κ*B and induced PGE_2_ production via COX-2 activation. As NF-kB and COX-2 are inflammatory mediators, the H&E staining after RFP administration was done. Results obtained showed that inflammation cell infiltration was observed, indicating that it was accompanied by tissue inflammation when RFP exerted purgative activity (Figure 3(d)). Moreover, RFP decreased the number of goblet cells (vacuoles) in enterocytes lining. This phenomenon might attribute to the production of PGE_2_, which was reported to promote the secretion of mucins from goblet cells, thus leading to the decrease of number of vacuoles in the colon [19, 20].

### 3.4. NF-*κ*B and COX-2 Inhibitors Suppressed RFP-Induced Laxative Effect

**Figure 4:**
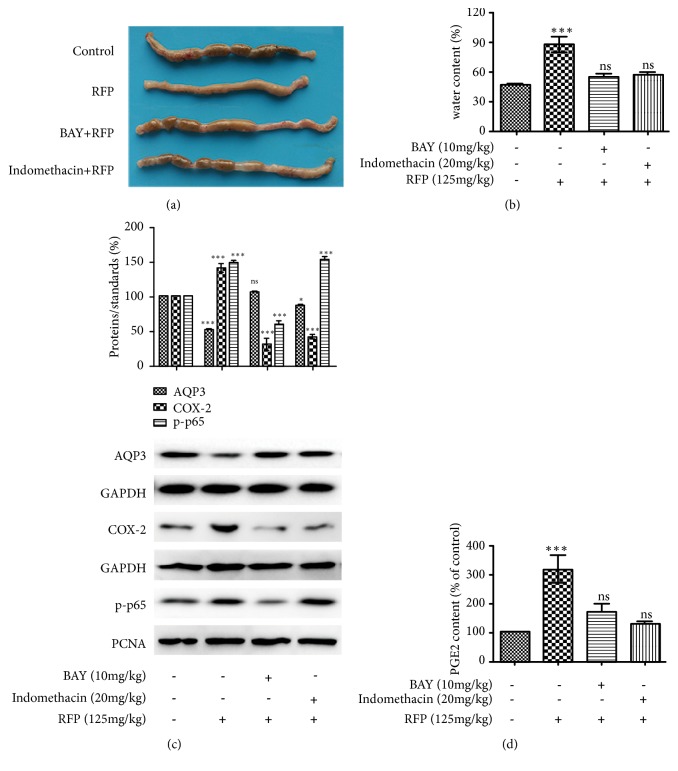
NF-*κ*B and COX-2 inhibitors suppressed RFP-induced laxative effect. (a, b, c, d) Pretreatment of rats by intraperitoneal injection of NF-*κ*B inhibitor BAY 11-7082 (BAY) (10 mg / kg) or COX-2 inhibitor indomethacin (20 mg/kg) for 1 h and further treatment with RFP (125 mg/kg). (a) After 6 h administration with RFP, rats were autopsied after sacrifice. Macroscopic image of the colon was observed. (b) Rat stool samples were collected 6 hours after RFP administration and fecal water content was measured, *∗∗∗*P < 0.001 or ns vs control group. (c) Protein expressions of AQP3, COX-2 and p-P65 were analyzed by western blot analysis. PCNA and GAPDH were used as an equal loading control. The data represented the means ± SDs for three experiments, *∗* P < 0.05, *∗∗∗*P < 0.001 or ns vs control group. (d) The levels of PGE_2_ were measured with ELISA methods and the control group was presented as 100%, *∗∗∗*P < 0.001 or ns vs control group.

To determine the role of NF-*κ*B and COX-2 in RFP-induced laxative effect, BAY 11-7082 (NF-*κ*B inhibitor) and indomethacin (COX-2 inhibitor) were applied. The feces in the colon of rats and the fecal water content after RFP administration were almost recovered to the normal level by BAY 11-7082 or indomethacin pretreatment (Figures 4(a) and 4(b)). Moreover, RFP-induced activation of COX-2 and production of PGE_2_ were effectively inhibited by BAY 11-7082 or indomethacin (Figures 4(c) and 4(d)). Interestingly, the RFP-induced activation of NF-*κ*B was suppressed by BAY 11-7082 but not indomethacin, suggesting that NF-*κ*B might be the upstream signal of COX-2. Additionally, the RFP-induced decrease in the expression of AQP3 was significantly inhibited by BAY 11-7082 or indomethacin. This observation may be ascribed to the inhibitory effect of NF-*κ*B and COX-2 by BAY 11-7082 or indomethacin, respectively.

### 3.5. RFP Decreased the Expression of AQP3 in HT-29 Cells

**Figure 5:**
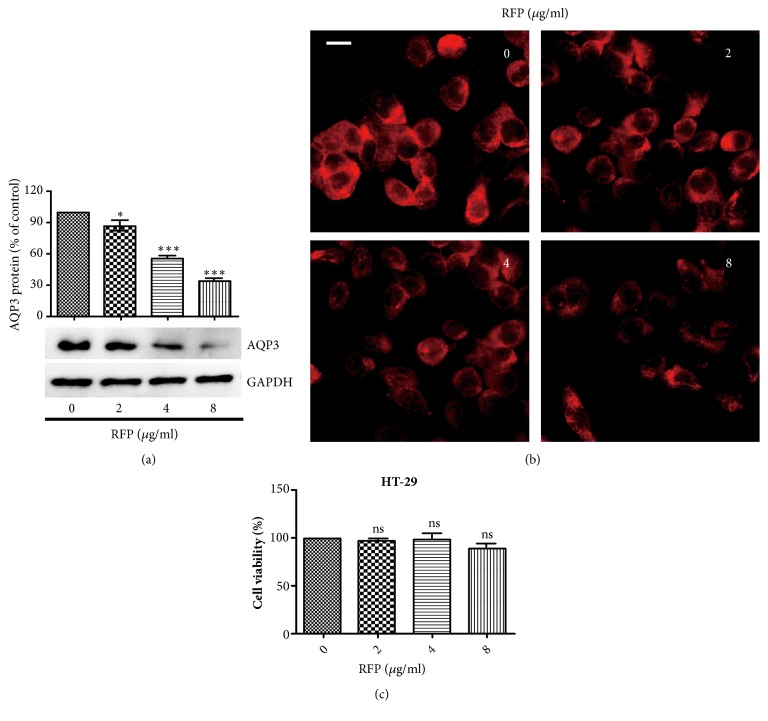
RFP decreased the expression of AQP3 in HT-29 cells. (a) Cells were lysed after treatment with RFP for 6 h. Western blotting was done to measure the protein expression level of AQP3. *∗* P < 0.05, *∗∗∗* P < 0.001 vs control group. (b) HT-29 cells were incubated with RFP (0, 2, 4, 8 *μ*g/ml) and then immunofluorescence assay was applied to observe the AQP3 expression (red) in HT-29 cells, bars: 10*μ*m. (c) Cells were treated with RFP at indicated concentrations for 6 h. The cellular viabilities were assessed by MTT. The data represented the means ± SDs for three experiments, ns vs control group.

To investigate the mechanisms of laxatives and diarrhea development, HT-29 cells which were derived from human colon cancer were used. The expression of AQP3 was suppressed in a concentration-dependent manner after RFP treatment for 6 h (Figure 5(a)). Similar to western blot analysis, immunofluorescence assay further confirmed that RFP decreased the expression of AQP3 (Figure 5(b)). Next, we examined whether the reduction effect of RFP is due to its cytotoxicity. There was almost no cytotoxicity on HT-29 cells after RFP (2-8 *μ*g/ml) treatment for 6 h under our present experimental conditions (Figure 5(c)), suggesting that RFP might explicitly decrease AQP3 expression in vitro.

### 3.6. RFP Activated NF-*κ*B Pathway and Their Downstream Proteins in HT-29 Cells

**Figure 6:**
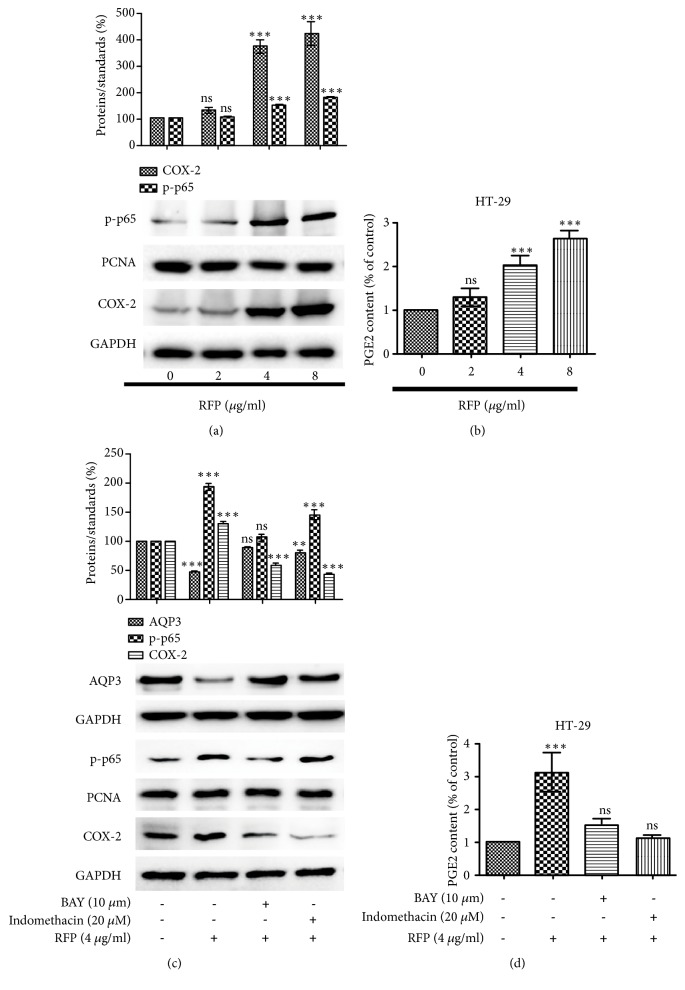
RFP activated NF-*κ*B pathway and their downstream proteins in HT-29 cells. (a, b) Indicated concentrations of RFP were applied to treat HT-29 cells. (a) Western blot analysed the transposition of p-p65 in the nuclear and cytoplasm COX-2. PCNA and GAPDH were used as an equal loading control. *∗∗∗*P < 0.001, ns vs control group. (b) PGE_2_ concentration was determined with ELISA kit, and the control cells presented as 100%. The data represented the means ± SDs for three experiments, *∗∗∗*P < 0.001, ns vs control group. (c, d) Cells were pretreated with NF-*κ*B inhibitor BAY 11-7082 (BAY) (10 *μ*M) or COX-2 inhibitor indomethacin (20 *μ*M) for 1 h and further treated with RFP (4 *μ*g/ml). (c) The expressions of p-p65, COX-2 and AQP3 were detected by western blotting. *∗∗*P < 0.01, *∗∗∗*P < 0.001, ns vs control group. (d) PGE_2_ concentration was measured with the same method above. The data represented the means ± SDs for three experiments, *∗∗∗*P < 0.001, ns vs control group.

Since NF-*κ*B was activated in vivo after RFP treatment, we investigated whether RFP could induce similar effects in HT-29 cells. Consistently in vivo, RFP caused a significant increase in the expression of NF-*κ*B and COX-2 as well as PGE_2_ production (Figures 6(a) and 6(b)). A further experiment was designed to test the role of NF-*κ*B and COX-2 in RFP-induced decrease expression of AQP3. Pretreatment cells with BAY 11-7082 or indomethacin before RFP stimulation, NF-*κ*B, and COX-2 as well as the production of PGE_2_ were inhibited while AQP3 protein level was significantly increased compared with RFP alone (Figures 6(c) and 6(d)). Results obtained suggested that inhibition of RFP-induced activation of NF-*κ*B or COX-2 could prevent the decrease of AQP3 expression in HT-29 cells.

### 3.7. PGE_*2*_ Decreased the Expression of AQP3 in HT-29 Cells

**Figure 7:**
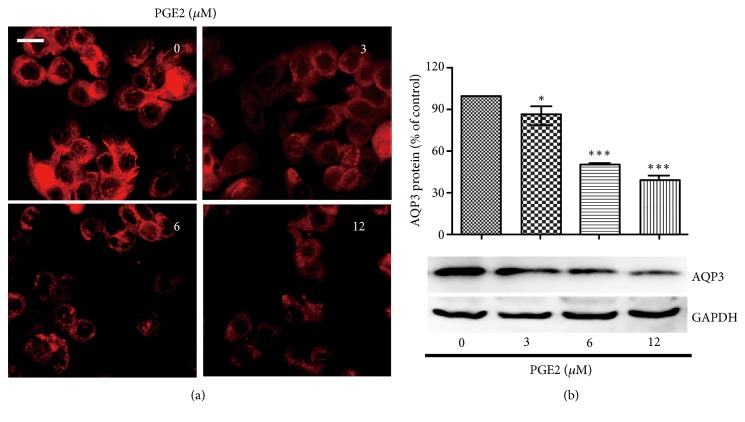
PGE_2_ decreased the expression of AQP3 in HT-29 cells. (a, b) HT-29 cells were treated with PGE_2_ with indicated concentrations for 6 h. (a) Immunofluorescence assay was applied to observe the expression of AQP3 (red) in HT-29 cells, bars: 10 *μ*m. (b) The expression of AQP3 was analysed by western blotting. *∗* P < 0.05, *∗∗∗* P < 0.001 vs control group.

It has been shown that PGE_2_ could induce the decrease expression of AQP3 [14]. Based on the fact that RFP could induce the production of PGE_2_, PGE_2_ was added to HT-29 cells. Fluorescence intensity results showed that the cell membrane and cytoplasm expression of AQP3 decreased significantly after PGE_2_ treatment for 6 h (Figure 7(a)). Western blot results further showed that PGE_2_ markedly reduced the expression level of AQP3 in HT-29 cells (Figure 7(b)).

## 4. Discussion

Recently, herbal plants have received increasing attention as new therapeutic drugs for the treatment of constipation and related diseases [21, 22]. In order to develop drugs for the treatment of constipation, we studied the therapeutic effect of Pharbitis Semen (Convolvulaceae), which is used as a traditional stimulant laxative herb in Korea, China, and Japan [23]. It is reported that resin glycosides are the material basis that account for the purgative action of convolvulaceous species as traditional purgative medicine throughout the world [8]. However, mechanism investigations on the laxative effect of resin glycosides from Pharbitis Semen have yet not yet been documented. In present study, we clarified the mechanism of the laxative effect of RFP. Here we have discovered and demonstrated the effects of RFP on AQP3 in vivo and in vitro. The main findings of our research are as follows: (1) RFP had the laxative effect via increasing water elimination in the colon. (2) RFP decreased the AQP3 protein expression in the colon of rats and HT-29 cells. (3) RFP activated the NF-*κ*B and COX-2 expression in vivo and in vitro; PGE_2_ acted as a performer to decrease the expression of AQP3.

Since tight junctions in colonic epithelial cells are rigid, AQPs play important roles in water transfer from the colon to the body [24]. AQPs 1-4 and AQP8 are found to be expressed in colon of animals [25, 26]. Among them, AQP3 has been extensively studied and is considered to function as a channel protein in dehydrating fecal contents [27, 28]. In this study, we found that RFP obviously decreased the AQP3 expression both in vivo and in vitro, which was confirmed by western blot analysis and immunofluorescence. In addition, a correlation was observed between a decrease in the expression level of AQP3 and an increase in the fecal water content. These results indicated that RFP administration may result in a decrease in AQP3 levels in colonic mucosal epithelial cells, which prevents water from reabsorption from the luminal side and eventually leads to diarrhea.

Furthermore, we explored the mechanism involved in RFP-induced decrease of AQP3 expression. In previous studies, NF-*κ*B activation is of great importance for the downregulation of AQP2 channel [26]. Another research reported that the COX-2 activation and PGE_2_ production are involved in the decrease expression of AQP3 [13]. Our investigations discovered that when NF-*κ*B or COX-2 activation was inhibited by pretreating rats with BAY 11-7082 or indomethacin, the laxative effect of RFP was alleviated and AQP3 protein expression almost recovered to the normal level in the colon (Figure 4). These results indicated NF-*κ*B or COX-2 activation might be involved in RFP-induced diarrhea. Additionally, we found that PGE_2_, which is the synthetic product of COX-2, increased significantly in a dose-dependent manner after RFP administration, revealing that PGE_2_ production might play an important role in RFP-induced diarrhea. This result was further confirmed by the inhibiting effect of BAY 11-7082 or indomethacin on PGE_2_ production when they relieved RFP-induced diarrhea.

In in vitro experiments, we examined the effect of RFP on HT-29 cells. Although they are derived from human colon cancer, they have been widely used to study the mechanism of diarrhea and laxatives due to the normal physiological state of the colon they represent [29]. Our examination showed that RFP administration to HT-29 cells significantly reduced the protein expression level of AQP3 and activated the NF-*κ*B pathway. Consistent to experiment in vivo, we found that RFP-induced decreased expression of AQP3 and increased production of PGE_2_ recovered to the normal level when pretreating cells with BAY 11-7082 or indomethacin. PGE_2_, as a downstream signal of NF-*κ*B, might directly regulate the expression of AQP3. Additionally, PGE_2_ was used to stimulate HT-29 cells; as a consequence, the expression of AQP3 was significantly downregulated. These results suggested the RFP-induced diarrhea was mediated by PGE_2_ secretion. Although the mechanism by which PGE_2_ reduces AQP3 expression remains unclear, it may increase endocytosis and degradation of AQP3.

NF-*κ*B is a quick responder of cellular responses because it is a primary transcription factor that can be activated by various stimuli without need for new protein synthesis [12]. In the present study, although RFP acting on NF-*κ*B directly or indirectly still needs to be confirmed, we found that RFP could activate NF-*κ*B, which was observed in vitro and in vivo. NF-*κ*B triggered the expression of COX-2 to accelerate the PGE_2_ secretion. PGE_2_ reduced the expression of AQP3, resulting in reduced water transport from intestine to blood vessel, leading to a laxative effect of RFP.

Pharbitis Semen was wildly prescribed for intractable constipation in many countries [9]. In this study, we found the presence of the inflammation response after the RFP-treated rats. Therefore, the dosage of administration and repeated administration of this purgative medicine should be noted.

## 5. Conclusions

In conclusion, the present study demonstrated that RFP induced its laxative effect by decreasing AQP3 expression, which was involved in water reabsorption from the intestinal tract to the vascular side in the colon. The RFP-induced decrease in the levels of AQP3 was confirmed in vivo and in vitro. Furthermore, we found that the decreased expression of AQP3 was caused by NF-*κ*B activation. NF-*κ*B triggered the expression of COX-2 to accelerate the secretion of PGE_2_, resulting in decrease in the expression of AQP3. These findings may explain the underlying diarrhea mechanisms of RFP and provide a sound basis for developing new therapy for constipation.

## Data Availability

The datasets analyzed during the current study are available from the corresponding author on reasonable request.
